# Inquiries Answered

**Published:** 1875-03

**Authors:** 


### INQUIRIES ANSWERED.

It would surprise our readers to know how frequently our advice is asked upon subjects scarcely connected with health or disease. Our old readers seem to take it for granted that we know nearly everything worth knowing, and that our opinion is valuable on other topics as well as on those to which our journal is mainly devoted. So we cften answer inquiries in these pages, without saying a word about the fact that such inquiry has been made. We do not waste space, which is so valuable, by putting the question in type; but we answer it, and those who have sent the inquiry know what it means.

Some weeks ago a friend wrote to learn the address of some excellent and honest furniture-dealer in New York city. We gave, in reply, the firm of DeGraffe & Cochrane, No. 152 West Twenty-Third Street, near Sixth Avenue. We could have given other good names, but none fulfilling every requisite. We were actuated in this matter precisely as we were during the past winter while being daily consulted with reference to the best heating device. We knew a good deal about furnaces and heaters, and we became convinced that the Gothic furnace of Lesley's was the best to be found. We said so from month to month, and hundreds of persons have thanked us for the information.

It is the same with reference to the inquiry of our subscriber about furniture. We know just what he seeks, for we know him to be a sensible man. He has built a new house on Long Island, and he wants to furnish it with that true economy which selects articles which will last a generation or more. He does not seek mere display—simple glitter. He wants grace and strength and honest workmanship combined. So we mention the house of DeGraffe & Cochrane; and feel that we have done our friend a service, while doing no injury to a house which is widely known for the excellent quality of its goods, its reasonable prices, and its fair and honest dealing. The advice which we cheerfully gave to one we as cordially repeat to all.

---

Over all life broods Poesy, like the calm blue sky, with its motherly rebuking face. She is the great reformer; and where the love of her is strong and healthy, wickedness and wrong can not long prevail.

